# Systematic detection of positive selection in the human-pathogen interactome and lasting effects on infectious disease susceptibility

**DOI:** 10.1371/journal.pone.0196676

**Published:** 2018-05-25

**Authors:** Erik Corona, Liuyang Wang, Dennis Ko, Chirag J. Patel

**Affiliations:** 1 Department of Biomedical Informatics, RTI International, Durham, NC, United States of America; 2 Department of Biomedical Informatics, Harvard Medical School, Boston, MA, United States of America; 3 Department of Molecular Genetics and Microbiology, Duke University Medical Center, Durham, NC, United States of America; 4 Department of Medicine, Duke University Medical Center, Durham, NC, United States of America; University of Alabama at Birmingham, UNITED STATES

## Abstract

Infectious disease has shaped the natural genetic diversity of humans throughout the world. A new approach to capture positive selection driven by pathogens would provide information regarding pathogen exposure in distinct human populations and the constantly evolving arms race between host and disease-causing agents. We created a human pathogen interaction database and used the integrated haplotype score (iHS) to detect recent positive selection in genes that interact with proteins from 26 different pathogens. We used the Human Genome Diversity Panel to identify specific populations harboring pathogen-interacting genes that have undergone positive selection. We found that human genes that interact with 9 pathogen species show evidence of recent positive selection. These pathogens are *Yersenia pestis*, human immunodeficiency virus (HIV) 1, Zaire ebolavirus, *Francisella tularensis*, dengue virus, human respiratory syncytial virus, measles virus, Rubella virus, and *Bacillus anthracis*. For HIV-1, GWAS demonstrate that some naturally selected variants in the host-pathogen protein interaction networks continue to have functional consequences for susceptibility to these pathogens. We show that selected human genes were enriched for HIV susceptibility variants (identified through GWAS), providing further support for the hypothesis that ancient humans were exposed to lentivirus pandemics. Human genes in the Italian, Miao, and Biaka Pygmy populations that interact with *Y*. *pestis* show significant signs of selection. These results reveal some of the genetic footprints created by pathogens in the human genome that may have left lasting marks on susceptibility to infectious disease.

## Introduction

Infectious disease is a major cause of death in every human population [1, 2]. Conditions especially favorable to transmission of infectious diseases emerged within the Neolithic era around ~10,000 B.C., as populations transitioned from the nomadic lifestyle to relatively permanent settlements. The urbanization that ensued caused a surge in the diversity and impact of disease for a variety of reasons [3–5]. The most infamous infectious disease outbreak is the Black Death pandemic that peaked in Europe during the mid-1300s. This pandemic was caused by the *Yersinia pestis* bacterium [6], which likely spread by rats and their fleas [7]. The Black Death killed 30–60% of the European population. Subsequent outbreaks were substantially less harmful [8], perhaps due to acquired immunity and genetic resistance to the disease.

Pathogens have been shown to contain constantly evolving genes. This characteristic confers the ability to remain virulent as immune systems and host genes themselves adapt over time [9–13]. Pathogens, like other environmental perturbations, have left their mark on human genomes [14–17] and it has been suggested that pathogens have been the main selective pressure throughout human evolution [16]. Karlsson et al. suggest the extreme death rate in diseases like the plague [8, 18] explains the presence of widespread unidentified selection signals in the human genome [19].

**Fig 1:**
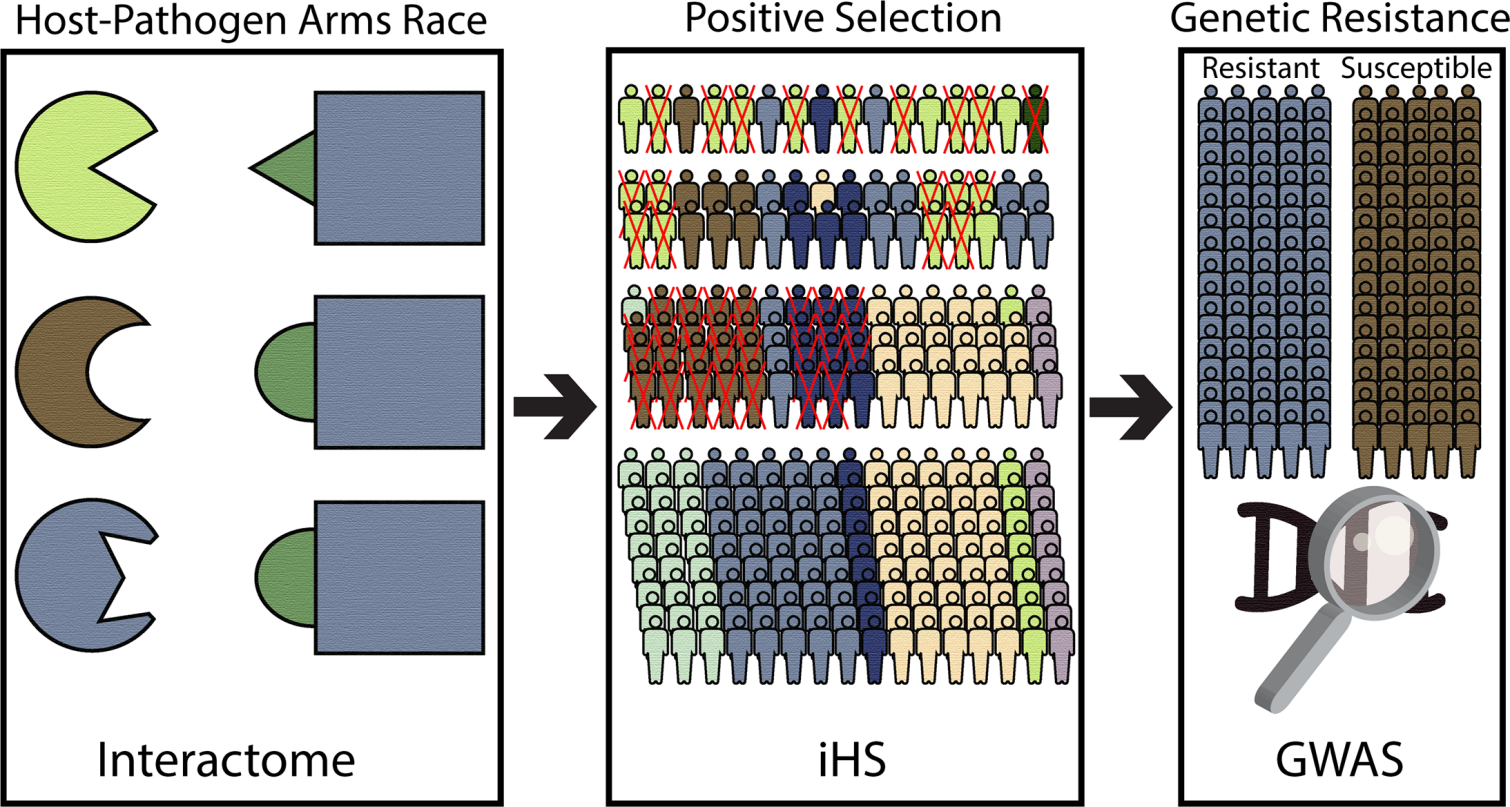
Host-pathogens arms race. A host-pathogens arms race can lead to significant modifications to the human genome of a host population over time. Human proteins that interact directly with pathogen proteins are often the target of strong positive selection. Random variation and novel mutations can naturally lead to increased fitness in certain individuals in a population with an endemic pathogen. Genetic resistance that has arisen due to positive selection acting on protective variants may be detected with a GWAS.

Beneficial alleles increase in frequency over time, and create haplotype structure perturbations that expose regions that have undergone *positive* selection. Haplotype-based positive selection methods have enabled the study of recent positive selection in the human genome [20, 21]. In contrast with methods relying on allele frequency or the number of nonsynonymous mutations, haplotype-based approaches excel at detecting selection during the Neolithic era [22], a time when infectious disease diversified and proliferated. However, haplotype approaches for studying natural selection are not designed to provide any causal information for selection events. They can be viewed as a single critical step in a larger approach to determine whether host-pathogen interactions have driven adaptive evolution in individual human populations. In this report, we address this challenge by integrating established methods for detecting haplotypes under natural selection with host-pathogen interaction data (Fig 1). We identify positive selection events that have acted on proteins that interact with pathogen proteins. Such modifications likely increased fitness in individuals within populations where a pathogen had a strong impact, as is the case with *Y*. *pestis*. Lingering genetic resistance could be identified using GWAS in cases where the selected variants are still protective and if the pathogen is active. In summary, our method links positive selection events with infectious disease in an effort to address the issue of widespread, yet unexplained signs of natural selection in the human genome.

We claim that a systematic attempt at detecting selection of individual human genes that interact with pathogens may shed light on how they have played a role in human adaptation. Interactions can include physical association, colocalization, and genetic interaction. Together, these interactions are referred to as the *interactome*. Viruses have been shown to be one of the most dominant drivers of evolutionary change in the part of the human proteome conserved within mammals [23]. Other studies on host pathogen interaction information have increased our pathophysiological understanding of infectious disease and have been used to characterize human proteins that interact with pathogens [24, 25], identify candidate disease genes [26], predict protein function [27, 28], create cross-species protein-protein interaction network alignments [29], and study pathogenesis of infectious disease [30, 31]. Positive selection has been identified in protein-protein interactions among loci associated with Alzheimer disease [32] and inflammatory disease [33]. We set out to identify individual pathogens that have impacted individual human populations. We achieved this goal by incorporating the human-pathogen interactome in order to systematically identify pathogens suspected of causing widespread signs of natural selection in individual human populations.

## Results

### Evidence of natural selection in host-pathogen interactomes

We examined the positive selection scores (iHS) of 53 populations in genes that interact with 26 different species of pathogens using the procedure shown in S1 Fig. We employed a resampling approach to determine when a set of proteins that interact with a particular pathogen exhibits more selection than expected by random chance. With a q-value cutoff of 0.05, we found evidence for positive selection in human genes that interact with proteins in the following 9 pathogens (out of 26 total queried), listed by increasing q-value for selection: *Yersinia pestis* (q-value = 5.62x10^-7^), human immunodeficiency virus 1 (q-value = 4.78x10^-5^), Zaire ebolavirus (q-value = 4.78x10^-5^), *Francisella tularensis* (q-value = 1.87x10^-4^), dengue virus (q-value = 1.87x10^-4^), human respiratory syncytial virus (q-value = 1.62x10^-3^), measles virus (q-value = 4.64x10^-3^), Rubella virus (q-value = 1.00x10^-2^), and *Bacillus anthracis* (q-value = 3.23x10^-2^). We replicated our analysis using African, European, and East Asian populations from HapMap Phase II. We were able to replicate our findings for *Y*. *pestis* (HapMap II European population p-value = 0.021) and for Measles (East Asian HapMap II population p-value = 0.016; S1 Table).

### Genes that interact with HIV-1 are under selection

**Fig 2:**
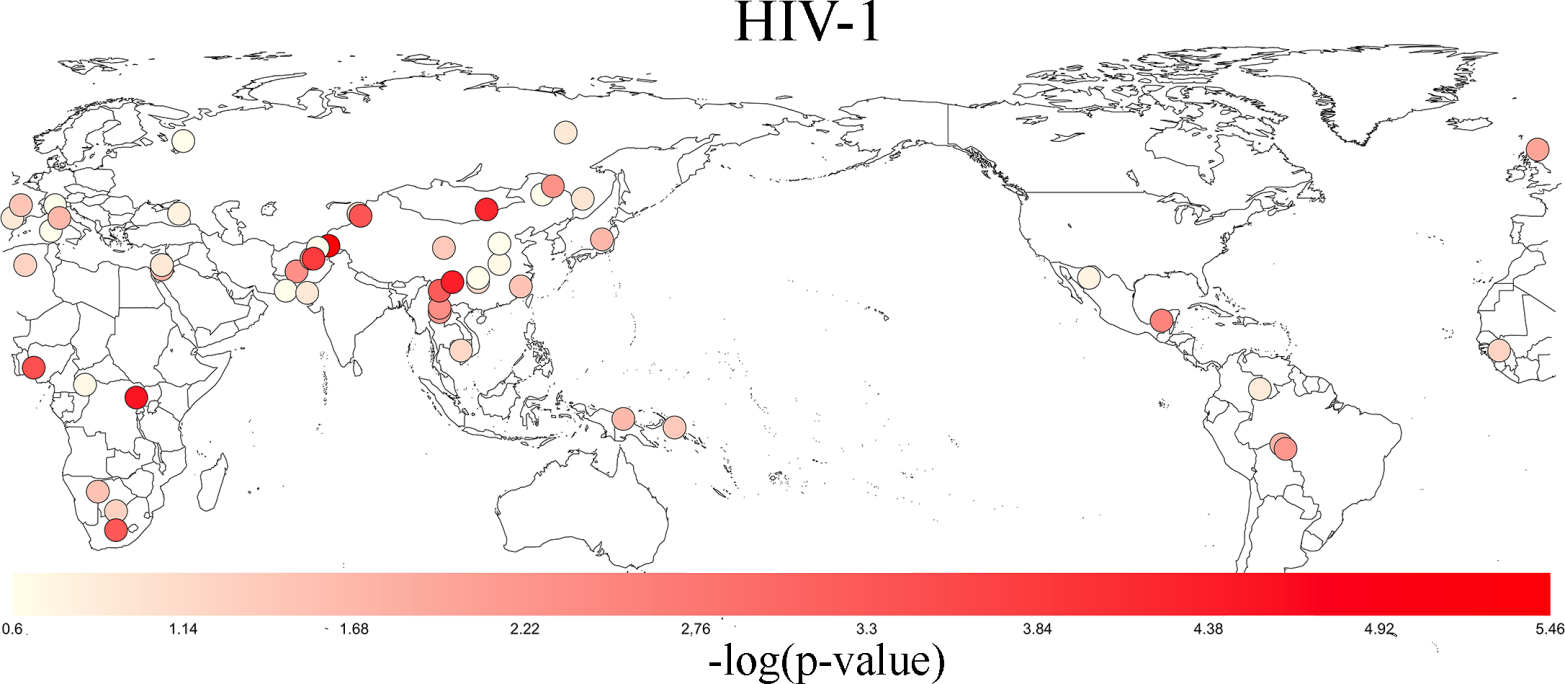
Worldwide levels of HIV-1 selection. A p-value for selection in human genes that interact with HIV-1 was produced for each population and is represented by a colored circle on the map. Significance is portrayed by a white to red gradient with lighter colors representing higher p-values and brighter red colors representing lower p-values. Only SNPs (filtered for LD) within genes that interact with Y. Pestis were included in determining the p-values. The most significant p-values were found in East Asia and Africa.

**Fig 3:**
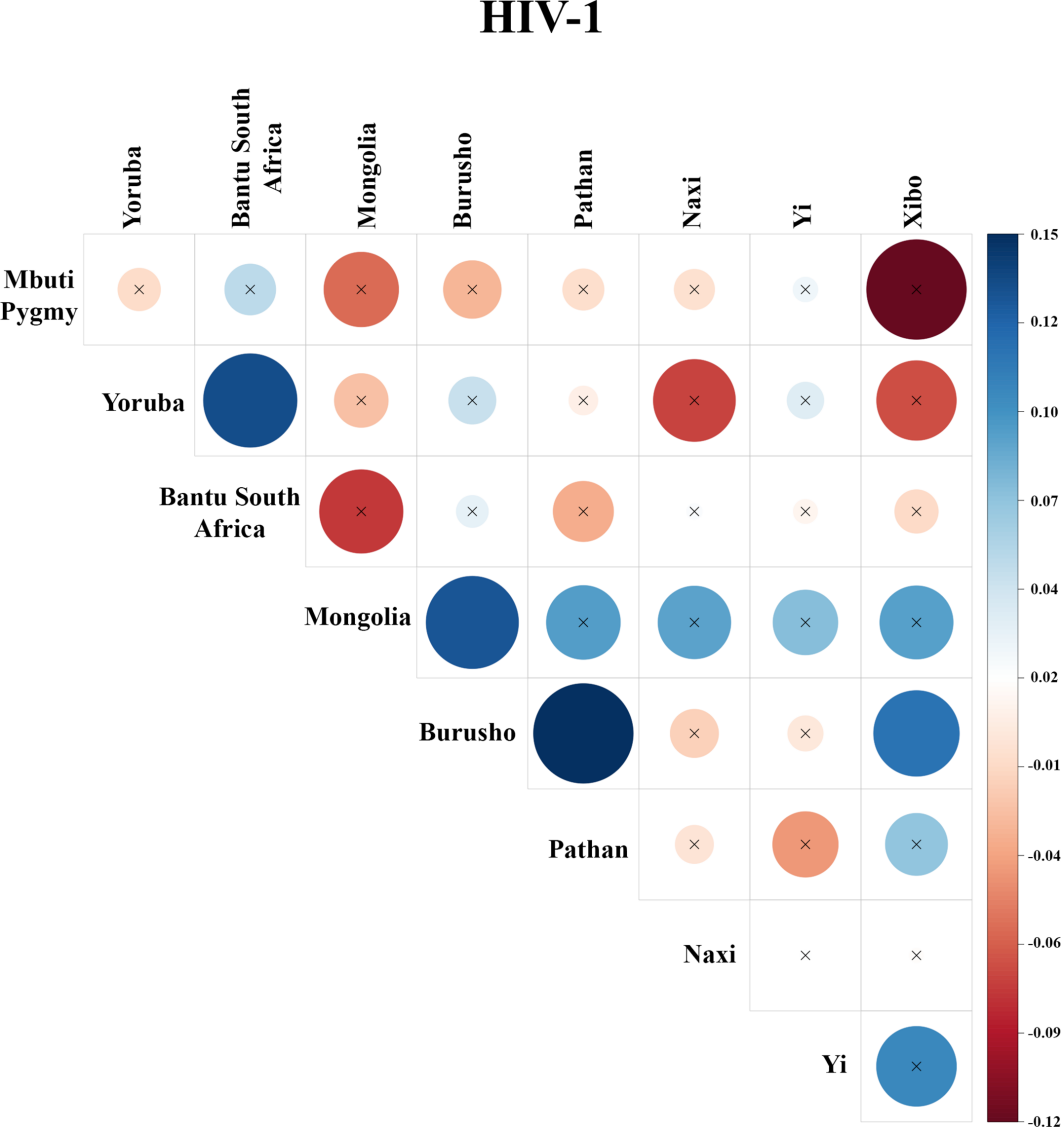
Overlap of selection targets associated with HIV-1. Each population was associated with a positive selection score for each gene interacting with Yersinia pestis. If gene scores were correlated for any population pair, there was significant overlap in the genes undergoing selection across both populations. We performed pairwise correlations among the 15 most selected populations to uncover the extent of overlap in selection targets. Kendall’s’ Tau-b coefficient ranged from -0.12 to 0.15. We identified 4 population pairs with significantly correlated positive selection scores.

There was evidence for positive selection associated with HIV-1, with a KS-test q-value of 4.78x10^-5^ (Table 1). Multiple human populations exhibit signs of positive selection in proteins that interact with HIV-1 (Fig 2), with the most significance detected in East Asia and Africa. The populations with the most significant signs of positive selection were Burusho, Mbuti, Yi, Mongolian, Pathan, Yoruba, Xibo, Bantu South African, and Naxi (p-value < 0.05, S2 Table). Five of these populations were East Asian, 2 were African, and 2 were Central South Asian. We investigated whether the same genes were under selection across these populations. Fig 3 shows pairwise population correlation coefficients produced with positive selection scores of genes that interact with HIV-1. There was significant, but modest, correlation in 4 population pairs. The largest Tau value of 0.15 is found between the Burusho and Pathan populations (one sided p-value of 2.64x10^-4^). This is perhaps not surprising given the geographic proximity of these two populations in modern-day Pakistan, but positive correlations were also detected for populations not in geographic proximity such as Burusho and Mongolia.

**Table 1:** Pathogen selection across worldwide populations.

#	Disease	Taxonomy ID	P-Value	Q-Value	Genes	Effect Size
1	Yersinia pestis	632	2.08x10^-8^	5.62x10^-7^	730	0.714
2	HIV-1	11676	3.70x10^-6^	4.78x10^-5^	373	0.712
3	Zaire Ebola virus	186538	5.31x10^-6^	4.78x10^-5^	6	0.869
4	Francisella tularensis	263	3.13x10^-5^	1.87x10^-4^	261	0.718
5	Dengue virus	12637	3.47x10^-5^	1.87x10^-4^	20	0.766
6	Human resp. syncytial virus	11250	3.61x10^-4^	1.62x10^-3^	46	0.742
7	Measles virus	11234	1.20x10^-3^	4.64x10^-3^	85	0.726
8	Rubella virus	11041	2.96x10^-3^	1.00x10^-2^	28	0.732
9	Bacillus anthracis	1392	1.04x10^-4^	3.23x10^-2^	493	0.700
10	Human herpesvirus 4	10376	2.87x10^-2^	7.76 x10^-2^	50	0.712
11	Human herpesvirus 8	37296	4.02x10^-2^	9.86x10^-2^	44	0.707
12	Human herpesvirus 1	10298	5.06x10^-2^	9.90x10^-2^	22	0.731
13	Human herpesvirus 5	10359	5.19x10^-2^	9.90x10^-2^	5	0.787
14	Sandfly fever Sicilian virus	28292	5.28x10^-2^	9.92x10^-2^	11	0.739
15	Vaccinia virus	10245	5.50x10^-2^	9.99x10^-2^	27	0.702
16	Hepatitis C virus	11103	6.65x10^-2^	0.101	243	0.702
17	Staphylococcus aureus	1280	8.32x10^-2^	0.121	7	0.712
18	Escherichia coli	562	0.120	0.168	28	0.695
19	California encephalitis virus	35305	0.134	0.179	13	0.716
20	Human mastadenovirus C	129951	0.637	0.777	9	0.693
21	West Nile virus	11082	0.655	0.779	10	0.674
22	Human adenovirus C	129951	0.637	0.789	9	0.703
23	Alphapapilloma virus 9	337041	0.762	0.866	6	0.655
24	Simian virus 40	10633	0.785	0.886	5	0.700
25	Influenza A virus	11320	0.886	0.945	107	0.692
26	Hepatitis B virus	10407	0.998	0.998	9	0.641

Human genes interacting with 26 pathogens were probed for signs of positive selection across 53 worldwide populations in the Human Genome Diversity Panel. A lower q-value indicates selection for the respective pathogen. The effect size of each disease is the mean of effect size across all populations.

**Fig 4:**
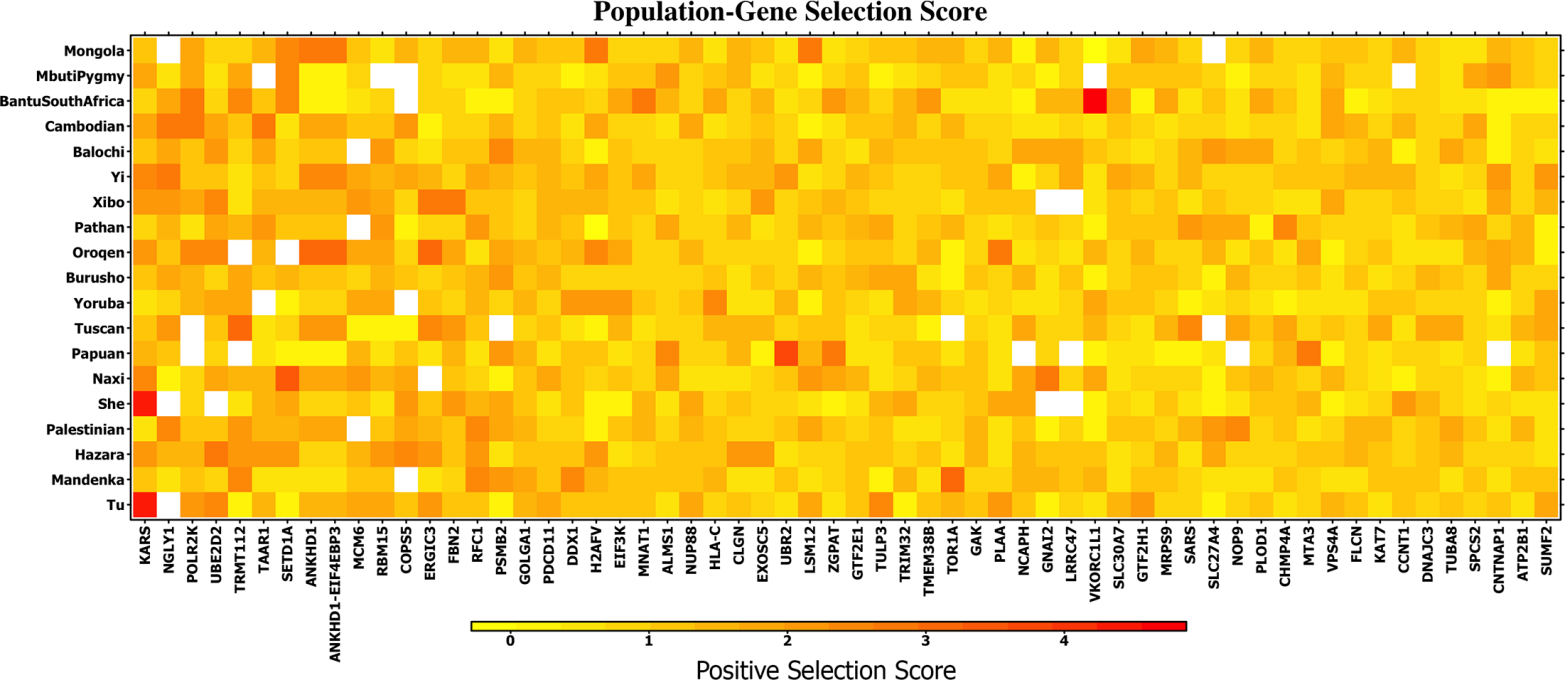
Genes that interact with HIV-1 under selection. The genes are sorted from left to right by highest to lowest mean selection across the 19 populations exhibiting a p-value < 0.1 for selection associated with HIV-1. The cumulative effect of a moderate selection signal allows for the detection of positive selection in these genes. The top 3 genes *are KARS*, *NGLY1*, and *POLR2K* (leftmost on the axis) are known inhibit HIV-1 replication.

We investigated whether human genes that interact with HIV-1 and have large positive selection scores (iHS score ≥ 2) were under selection in multiple populations. Fig 4 shows scores of the most selected genes among the 19 populations displaying the most significant signs of selection. The genes on the x-axis are sorted by decreasing mean selection scores for the populations shown. The gene with the largest mean positive selection score was *KARS*. It has undergone recent positive selection in the East Asian populations Tu and She with scores 4.96 and 4.50, respectively. Knockdown of this gene by siRNA inhibits the early stages of HIV-1 replication [25]. *NGLY1* is the second most selected gene and it also inhibits HIV-1 replication in some cells [34] as does *POLR2K*, which is the third most selected gene [35].

We examined published GWAS studies of HIV risk and progression, to determine if genes in the HIV-human interactome were (1) enriched with GWAS associations and (2) if that enrichment increased with increasing evidence of natural selection. Eight studies [36–43] from the GRASP GWAS Catalog [44] examining HIV susceptibility and host response were queried to obtain 2502 SNPs that showed evidence of association with susceptibility, host control, and progression of HIV infection. Using a p < 1x10^-5^ threshold for GWAS SNP inclusion, we saw a moderate enrichment of human-HIV interactome SNPs (p = 0.07, 3.0 fold-enrichment; Table 2). The fold-enrichment and the statistical significance progressively increased when we restricted to more stringent p-value (GWAS p < 1 x 10^−10^) and iHS thresholds (iHS > 4) (p = 0.001, 37.2 fold-enrichment). SNPs in/near human genes that interact with HIV genes are more likely to be associated with HIV susceptibility and outcome, and this enrichment is greater for SNPs exhibiting evidence of natural selection.

**Table 2:** Overrepresentation of HIV human interactome genes in GWASes.

	P < 1e-5		P < 1e-10	
	# of shared SNPs	Fold enrichment	# of shared SNPs	Fold enrichment
iHS > 0	3	3.03	2	3.84
iHS > 2	3	3.37	2	4.27
iHS > 3	2	4.33	2	8.22*
iHS > 4	2	19.57**	2	37.17**

SNPs in human genes in the HIV interactome and under natural selection are enriched in GWAS of HIV susceptibility/progression. P value thresholds for HIV GWAS and for iHS thresholds are indicated. Fisher’s exact test was used to calculate the significance of the enrichment.

* = p < 0.05.

** = p< 0.01

### Selection in genes that interact with *Yersinia pestis*

**Fig 5:**
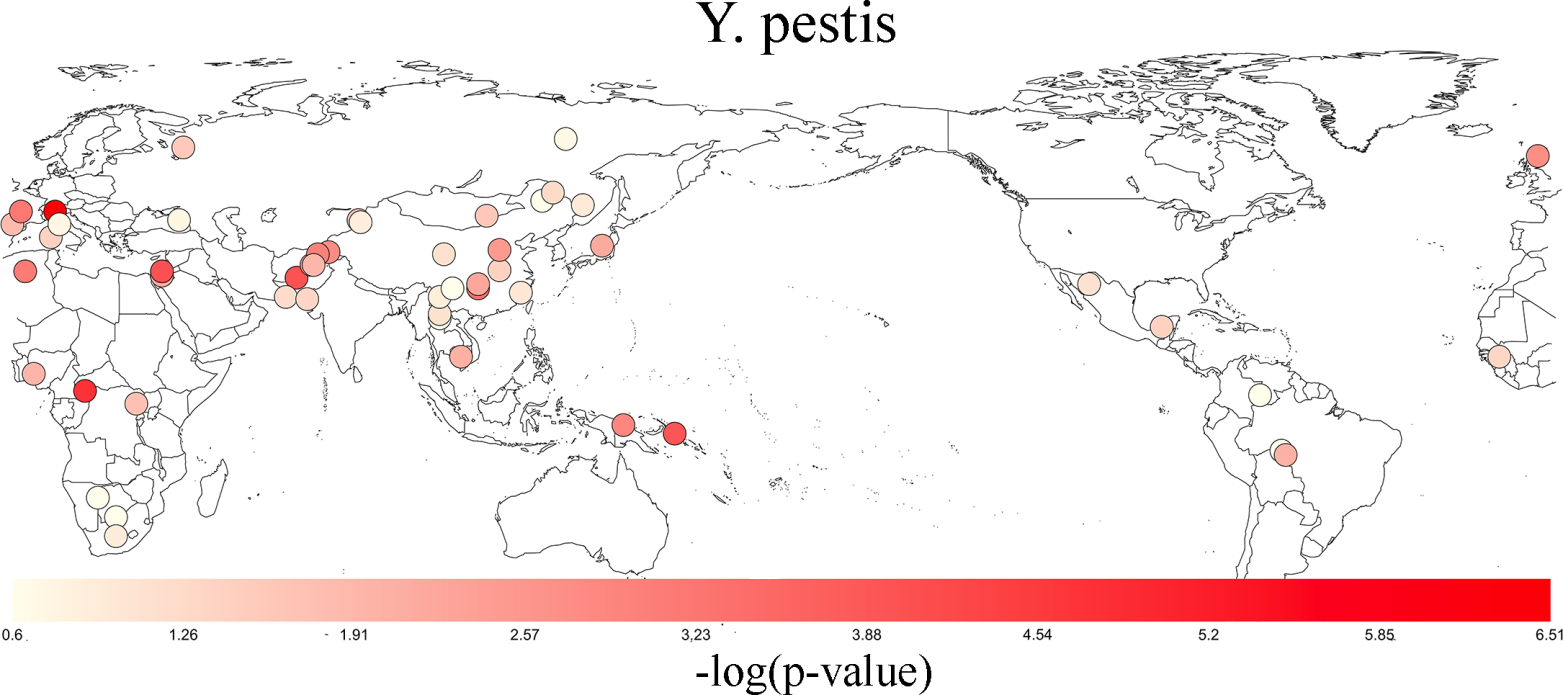
Worldwide selection of Yersinia pestis. A p-value for selection in human genes that interact with Yersinia pestis was produced for each population. Each population is represented by a colored circle on the map. The significance for selection is portrayed by a white to red gradient with lighter colors representing higher p-values and brighter red colors representing lower p-values. Only SNPs within genes that interact with Yersinia pestis were included in determining the p-values of selection for each population. These SNPs were filtered for LD. The most significant p-values were found in Europe, Central South Asia, and Africa.

**Fig 6:**
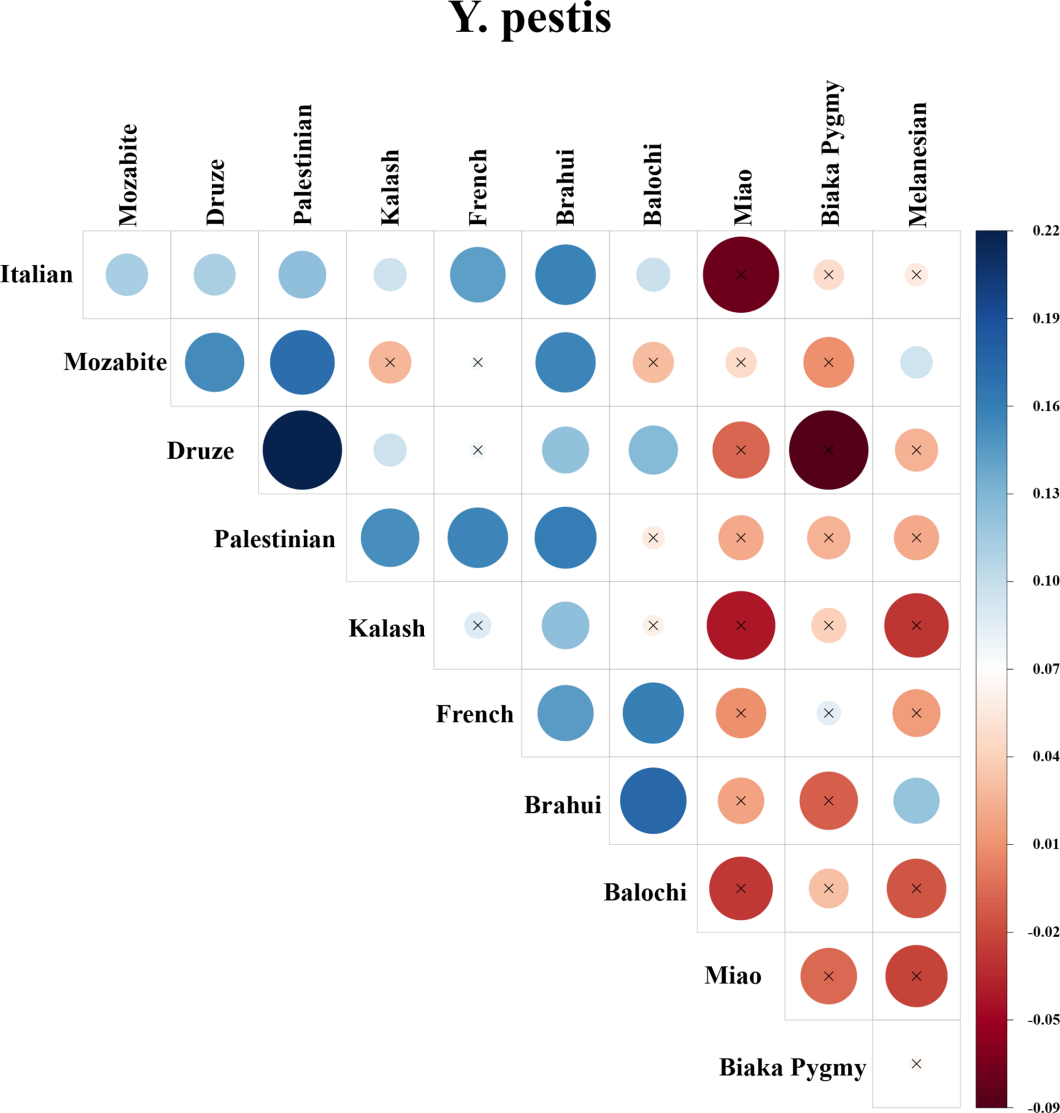
Overlap of selection targets associated with Yersinia pestis. Each population was associated with a positive selection score for each gene interacting with Yersinia pestis. There was significant overlap in the genes undergoing selection across both populations in many populations. We performed pairwise correlations among the 11 most selected populations to uncover the extent of overlap in selection targets. Kendall’s’ Tau-b coefficient ranged from -0.09 to 0.22. We identified 23 population pairs with significantly correlated positive selection scores (a positive selection score is produced for each gene).

**Fig 7:**
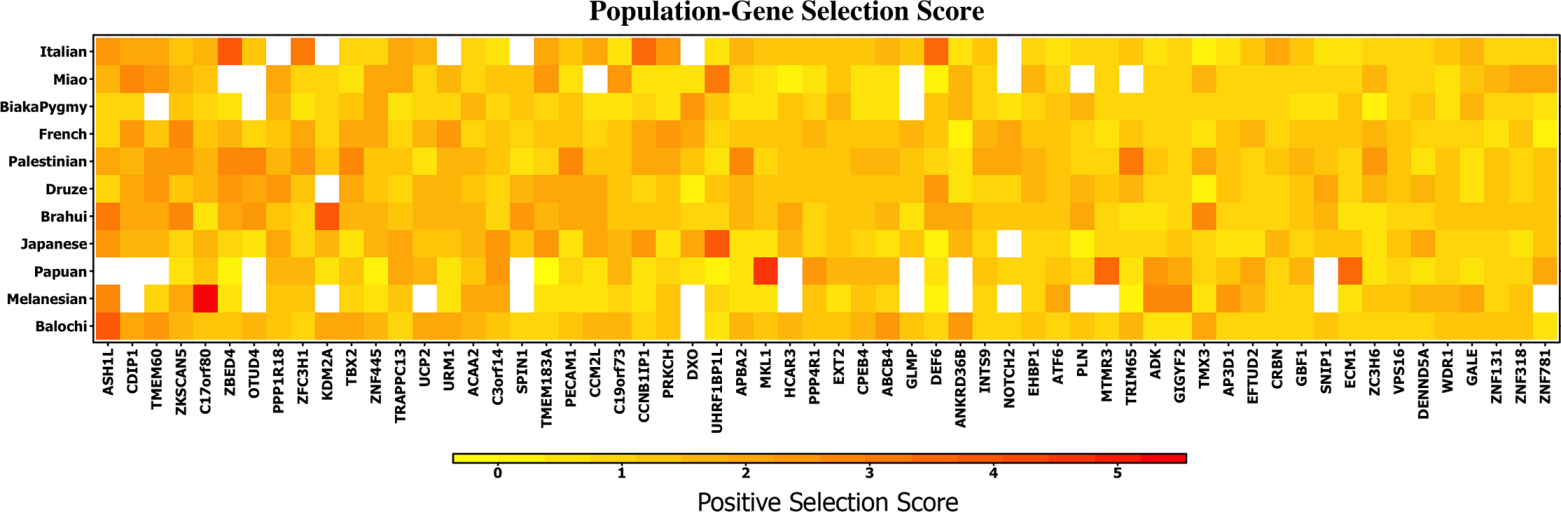
Human proteins that interact with Y. Pestis proteins under selection. The genes are sorted from left to right by highest to lowest mean selection across the 9 populations represented. Only populations with a p-value < 0.1 for selection in proteins that interact with HIV-1 were included in this figure. Selection is primarily driven by moderately selected genes. Genes such as *ASH1L*, *CDIP1*, *TMEM60*, and *ZKSCAN5* exhibit strong selection across multiple populations. Genes with the most significant p-values for selection are shown from left to right. Some genes do no exhibit a positive selection score (|iHS|> 2) in any population, indicating that positive selection is detected only by enrichment as no single population exhibits strong selection for such genes.

There was evidence for positive selection with *Y*. *pestis*, with a q-value of 5.62x10^-7^ generated by examining 53 worldwide populations. Fig 5 is a map showing the location of each population studied and the population’s positive selection p-value. The most significant p-values were found in Europe and Asia (though not exclusively so). Table 3 shows the most significant p-values for positive selection are associated with Italian, Druze, Biaka Pygmy, Palestinian, and Brahui populations. We investigated whether the same genes exhibited positive selection across multiple populations. To accomplish this, we tested for correlation of positive selection scores in genes that interact with this pathogen. The positive selection scores for these genes were significantly correlated across multiple population pairs, and the maximum Kendall’s tau coefficient observed was 0.22. Fig 6 shows pairwise correlation coefficients. Significant correlation was detected among multiple populations, indicating that there is overlap in functional processes under selection in the human genome across even distantly related populations. As shown in Fig 7, the observed signal for selection is being driven by individual genes that exhibit strong selection in single populations (e.g. *C17orf80* and *MKL1*), as well as genes that show consistent selection across many populations as is the case with *CDIP1*, *ZNF445*, and *URM1*.

**Table 3:** *Y*. *Pestis* selection across worldwide populations.

#	Region	Population	Effect Size	P-Value	SNPs
1	Europe	Italian	0.760	1.49x10^-3^	442
2	Middle East	Druze	0.752	7.59x10^-3^	456
3	Africa	Biaka Pygmy	0.734	8.74x10^-3^	449
4	Middle East	Palestinian	0.743	1.79x10^-2^	452
5	Central South Asia	Brahui	0.739	1.81x10^-2^	453
6	East Asia	Melanesian	0.746	1.94x10^-2^	416
7	Oceania	Balochi	0.732	3.16x10^-2^	457
8	East Asia	Miao	0.726	3.30x10^-2^	438
9	Europe	French	0.738	4.12x10^-2^	459
10	Middle East	Mozabite	0.733	4.43x10^-2^	458
11	Central South Asia	Kalash	0.731	4.98x10^-2^	451
12	Oceania	Papuan	0.736	5.50x10^-2^	423
13	Central South Asia	Burusho	0.726	6.23x10^-2^	461
14	Europe	Orcadian	0.731	6.58x10^-2^	446
15	Central South Asia	Hazara	0.728	7.82x10^-2^	454
16	East Asia	Han	0.723	8.15x10^-2^	431
17	East Asia	Tujia	0.713	0.107	434
18	Middle East	Bedouin	0.719	0.113	453
19	East Asia	Japanese	0.732	0.114	443
20	East Asia	Cambodian	0.709	0.130	432

Populations are sorted in descending order of evidence for positive selection with respect to *Yersinia pestis*. The effect size represents the mean |iHS| across all SNPs in genes that interact with *Yersinia pestis*.

## Discussion

This study systematically investigated the human-pathogen interactome for signs of positive selection within the human genome using a haplotype based positive selection detection method. Host-pathogen protein-protein interactions may have caused widespread positive selection signals detectable in present day populations and these selected variants may confer genetic resistant against pathogens to this day (Fig 1).

We have identified positive selection in human genes that interact with 9 different pathogens across multiple worldwide populations (Table 1). Our results suggest that these (or closely related) pathogens may be responsible for the observed signals of natural selection. Importantly, we also observed that selected loci, for the case of HIV, were overrepresented for susceptibility variants as elucidated by GWAS. Specifically, enrichment analysis of HIV susceptibility GWAS demonstrated SNPs in HIV interactome genes were more likely to be associated with HIV susceptibility and host control, and the level of enrichment increased for genes that also demonstrated evidence of positive selection. For *Y*. *pestis*, no GWAS of bubonic plague exists to demonstrate a similar enrichment with *Y*. *pestis* interactome genes. We have developed an approach for studying natural selection in humans connecting the drivers of natural selection (host-pathogen interactions), to the signatures they leave behind (long haplotypes), to their lasting impact on disease susceptibility (genetic association with risk).

We probed human genes interacting with 26 pathogens for signs of positive selection in 53 worldwide populations. Our method is suitable for identifying pathogens that have caused haplotype structure perturbations in worldwide populations. We uncovered evidence of positive selection associated with 9 of the 26 pathogens studied. Our study reveals that probing all genes that interact with a pathogen for signs of positive selection can identify pathogens that may have altered the human genome. We also showed that while there is some overlap in the functionality that has most likely undergone selection as a response to pathogen exposure, there is also great diversity in the genes that have undergone selection across separate human populations. We speculate that many of the present day populations used for this study may have increased genetic resistance against specific pathogens due to past exposures.

### HIV-1

We detected positive selection in human genes that interact with HIV-1. The detection of positive selection associated with HIV-1 may at first seem surprising, due to the fact that this disease emerged in humans during the first half of the 20^th^ century [45]. Thus, not enough time has passed since HIV emerged in humans for positive selection to be detected with the iHS method. Our results support the hypothesis that humans have been repeatedly infected with lentiviruses like HIV-1 [19, 46]. Karlsson et al., report that humans, particularly those in Africa, are likely to have experienced ancient lentivirus epidemics. There were 10 documented cases of cross-species transmissions events in humans [47] in the last century alone. It’s plausible that countless other zoonotic transmissions of this sort have occurred multiple times throughout human evolution.

This study supports the hypothesis that lentivirus epidemics occurred elsewhere, including East and Central-South Asia. Specifically, the Burusho, Yi, Mongolian, Pathan, Yoruba, and Xibo populations show signs of positive selection (p-value < 0.05; S2 Table and Fig 2). As SNPs in genes within the HIV interactome show an enrichment in GWAS data for HIV susceptibility, common genetic variation in these genes appear to continue to regulate HIV infection and outcome. This enrichment is driven by SNPs in MHC class I genes (*HLA-B*, rs1058026 and *HLA-C*, rs13207315), as shown in Table 2. While the ubiquitous importance of MHC in infectious disease makes it difficult to discern whether the natural selection detected at these genes are specifically due to past lentiviral pandemics, they clearly have functional relevance for HIV infection.

The top 3 most selected as shown in Fig 4 are *KARS*, *NGLY1*, and *POLR2K*. These genes are known to inhibit HIV-1 replication. Other genes in this list such as *TAAR1* have no *known* antiviral activity, but may be protective against HIV-1 due to consistent selection across multiple populations. Genes under strong positive selection were not necessarily selected in multiple populations (i.e. different human genes that interact with HIV-1 underwent selection across separate populations). This showcases the diverse, yet consistent pattern of positive selection associated with HIV-1 that emerges when viewed across multiple worldwide populations.

Despite the occurrence of individual human proteins undergoing selection in primarily a single population, we addressed whether proteins exhibit a positive correlation across distantly related populations. Populations that are distantly related such as Burusho and Xibo have been separated for such a long period of time that selection signals could not have been inherited from a common ancestor. Fig 3 shows that the Burusho and Xibo populations have a statistically significant correlation in the proteins that interact with HIV-1. This serves as evidence for convergent evolution between these two populations that may have been induced by an ancient lentivirus like HIV-1.

### Yersinia pestis

*Y*. *pestis* is one of the most deadly human pathogens [8, 18]. It is reasonable to expect that infection with it caused major evolutionary perturbations in the genomes of previously infected populations. Consistent with previous studies [48, 49], we have found that genes interacting with this pathogen exhibit positive selection in multiple populations in Europe, East Asia, Africa, and the Middle East (Table 3). The plague has affected multiple worldwide populations [50]. We postulate that the high death rate and virulence associated with *Y*. *pestis* caused positive selection that can be detected in multiple present day populations in the *Y*. *pestis* and human interaction network.

The positive selection scores for genes that interact with *Y*. *pestis* were modestly correlated across distantly related populations. Populations such as the Brahui and Melanesian populations are distantly related, yet exhibit significant correlation of positive selection scores in proteins that interact with *Y*. *pestis* (Fig 6). Such distantly related populations underwent *de novo* parallel positive selection for an overlapping set of genes (i.e. convergent evolution) as they are too distantly related for the positive selection signal to have originated in the genome of a common ancestor. Our analysis suggests that mutations of the same genes in multiple populations conferred genetic resistance against *Y*. *pestis*. This figure also shows that a different set of genes underwent selection across different populations. This presents an opportunity to learn more about functional components that underwent selection in response to *Y*. *pestis*.

We investigated if the genes exhibiting the largest positive selection scores were under selection in an individual population or in multiple populations at once. Populations exhibiting moderate signs of selection (populations with p-value < 0.1 in Table 3) are shown in Fig 7. These populations were used to compute the mean score across all proteins that interact with *Y*. *pestis* proteins. The genes are ordered from left to right, starting with the most selected to least selected genes. Some genes like *ASH1L* exhibit consistent moderate selection across populations while other genes like *C17orf80* exhibit strong selection in primarily the Melanesian population. We examined genes with iHS scores of ≥ 3 in order to derive insight into those that have likely undergone positive selection. These genes are *ASH1L*, *C17org80*, *ZBED5*, *KDM2A*, *UHRF1BP1L*, *MKL1*, *DEF6*, and *MTMR3*. The gene *UHRF1BP1L* has undergone recent selection in the Japanese and Miao populations with scores of 3.36 and 3.17, respectively. This gene’s product is associated with the cell cycle and cellular proliferation in multiple cancers [51–56]. Downregulation of *UHRF1BP1L* causes G2/M arrest, activation of DNA damage response, and apoptosis [57]. The gene *OTUD4* has undergone positive selection in 3 populations; Palestinian (score: 2.62), Brahui (2.28), and Druze (2.13). Little is known about *OTUD4*’s function. Its product contains a cysteine protease domain found in viruses, eukaryotes, and *Chlamydia pneumoniae*. It has a smaller alternatively spliced isoform found only in HIV-1 infected cells [58].

### HapMap Phase II replication

We replicated our analysis with the HapMap Phase II cohort consisting of 3.1 million SNPs in 3 different populations. It is unlikely that widespread replication would be observed because the HGDP data contains 53 populations versus the 3 available in the HapMap Phase II cohort. This study succeeded in detecting widespread selection when the signal of selection was combined across multiple populations, which is not possible with the 3 HapMap phase II populations. We did expect to find some replication and indeed we found evidence for selection for 3 of the 9 pathogens detected in the HGDP data (S1 Table).

### Other pathogens

We detected selection in several other additional pathogens. The data suggest that these pathogens may have caused ancient pandemics in several populations. Some of these diseases have relatively high morbidity rates even today. For example, nearly one hundred percent of all children are infected with respiratory syncytial virus (RSV) by the time they are 3 years old [59, 60]. This is in contrast to *Bacillus anthracis*, the bacterium that causes anthrax. Until the 20^th^ century, anthrax killed hundreds of thousands of animals and people each year, but its incidence rate has diminished and cases are now rare.

### Limitations

Protein-protein interaction databases contain a significant number of false positive interactions. For example, protein interactions found in yeast cells via yeast-two hybrid library screening may not actually occur in an organism: the proteins may be expressed in different tissues or at different times and may not encounter each other. In addition, errors may be introduced during manual data curation. False positives are unlikely to bias our findings because they add noise to data when attempting to detect positive selection. This would require the signal to be stronger in order to detect selection associated with a pathogen’s interaction network. We leveraged the iHS method which detects differences in LD associated with different alleles on the same SNP in order to detect selection. This approach is well-suited to identify recent selection sweeps that take an allele from a low frequency to a high frequency. Its sensitivity decreases as the age of an allele and its population frequency increase, because LD disparities become less pronounced [61]. This analysis will fail to detect positive selection of old variants (> 25,000 years old) that protect against infectious disease.

The number of infectious organisms tested for selection is only a small fraction of all pathogens. It is possible that similar species have overlapping interaction networks, causing selection to be reported for one pathogen even if it was caused by a closely related pathogen. This possibility is further complicated by the fact that the rate of protein-protein interaction evolution may be three orders of magnitude lower than the rate of protein sequence evolution [62]. The inclusion of as many infectious organisms as possible would increase the likelihood that the causal pathogen has been identified with our approach. In addition, we based our analysis on genes that interact exclusively with a single pathogen which excluded a large number of genes from this study that are potentially important to the process of genetic resistance. This does not interfere with the overall goal, which is to detect selection in genes that interact with specific pathogens rather than detect genes that have undergone selection.

### Conclusions

We have identified specific pathogens that demonstrate evidence of natural selection in human populations. Our work uncovers specific populations that have likely been exposed to the plague, lentiviruses, and various other diseases. Populations that display positive selection in genes that interact with pathogens likely have inherited some level of resistance against the causal pathogen. Further work could include testing whether such populations have decreased risk or severity of infections resulting from such pathogens. A database containing medical health records along with genetic data for patients should facilitate testing this hypothesis. It is also possible to identify specific variants that have undergone positive selection and test whether individuals with such variants are more successful in fighting the associated infectious organisms as a complementary strategy to GWAS. Here, we have identified a large set of populations that have likely undergone selection after pathogen exposure, and have produced a set of genes that exhibit strong signs of selection. Future work will focus on identifying protective variants within these genes to elucidate causal relationships between pathogen resistance and adaptation. The identification of such variants could provide further data to predict infectious disease outcomes based on genome data for patients.

## Methods

### Data

We investigated SNPs from the Human Genome Diversity Panel (HGDP), which consists of >650,000 samples from 53 populations on 8 continents [63]. Each population was probed for selection. We also used the HapMap Phase 2 cohort (3.1 million SNPs) to analyze African, European, and Asian populations [64]. Human-pathogen interaction data was obtained by combining data in BioGrid 3.2 [65], IntAct [66], and VirusMint [67] as of January 14, 2014. [67–69]. These databases contain a large number of curated human-pathogen interactions discovered by methods including tandem affinity purification, yeast two hybrid assays, coimmunoprecipitation, and phage display.

### Detection of positive selection with the integrated haplotype score

We used the integrated Haplotype Score (iHS) to detect positive selection in human genes that code for proteins that interact with pathogen proteins [21]. The iHS relies on haplotype structure to detect positive selection. It does so by identifying haplotype structure differences between two alleles in a SNP. Positive selection pressure applied to a low frequency allele will cause an increase in haplotype homozygosity (the number of identical haplotype blocks in the region). This represents an overall decrease of diversity, but only in the haplotype blocks linked to the selected allele. Eventually, recombination and mutations will make this haplotype block perturbation increasingly difficult to detect. Each iHS has a positive or negative sign, depending on whether selection pressure was applied to the ancestral or derived allele. We used absolute values of the iHS, as our goal was to identify indications of selection, irrespective of whether the target was ancestral or derived.

A useful characteristic of the iHS is that, when mean-subtracted and divided by the standard deviation, scores are roughly normally distributed with mean 0 and variance 1. Because we used the absolute values of each iHS, their theoretical distribution is a folded normal distribution with a mean of $\sqrt{\frac{2}{\pi }}$ and a variance of 1–2/ π. Under the assumption that this distribution represents the iHS, the mean score of any number of SNPs will always be $\sqrt{\frac{2}{\pi }}$. However, deviations from this distribution occur because genic regions are more conserved than non-genic regions and the iHS undergoes z-score normalization across all SNPs. This fact explains the deviation from the observed folded normal distribution, but it did not affect our results because we used a non-parametric resampling approach towards detecting selection in a collection of iHSs. Finally, iHSs for the HGDP and HapMap Phase II populations computed in previous projects [21, 70] were integrated into this study.

### Positive selection score for a pathogen in a population

The human pathogen interactome was used to identify human genes whose products interact with pathogens. All organisms of the same species were grouped and analyzed as a single organism. The set of human genes that interacted with a pathogen was used to represent an infectious disease. All SNPs in these genes were used to detect positive selection among human genes. We removed all human genes that interacted with more than one pathogen to ensure specificity in host-pathogen interactions. We also used a conservative filtering method to remove SNPs in linkage disequilibrium (LD); this process ensured that all iHS scores were independent. Our method for producing a positive selection score for a pathogen in a single population is as follows (see also Part A in S1 Fig). First, we created an interaction database by combining data from multiple sources. In step 2, we identified human genes that interacted exclusively with a target pathogen. Step 3 was to identify all SNPs within 0.5kb (3’) and 2kb (5’) of the human genes that interact with a pathogen. Step 4 was to add the iHS computed in the target population to each SNP identified in Step 3. In step 5, we filtered for LD, which led to an independent set of SNPs with iHS scores representing selection of a pathogen in the human genome. These scores were summed in step 6 to produce a single value that represented a measure of selection associated with the pathogen in the target population. The mean of these SNPs represented the selection effect size for the pathogen in the target population. It was used to compare the relative impact of selection for the pathogen in different populations.

The positive selection scores of SNPs in LD are not independent. A selective sweep will cause the iHSs to be high for many SNPs surrounding the selected SNP. We filtered for LD in order to include only a single SNP within a region caught in a selective sweep. Otherwise, genes with larger SNP densities will appear to have undergone positive selection as moderate iHSs would have a cumulatively large effect. Step 5 in Part in A S1 Fig corresponds to the steps in Part C in S1 Fig. We expanded on the process to filter SNPs in LD. We start by taking all SNPs that interacted with the target pathogen (set A). This includes SNPs that are in LD and exhibit correlated iHSs. Our first step to attain a representative positive selection score across all correlated iHSs was to remove the SNP with the median iHS. The SNP was then added to a set of “independent” SNPs that interact with the target pathogen (Set B), which started as an empty set. The second step eliminates all SNPs within 1Mb of the removed median SNP from set A, ensuring that SNPs with correlated iHSs to the median SNP are removed. Steps 1 and 2 were repeated until there were no more SNPs in set A. Each “median” SNP removed from set A represents a 2Mb region.

These steps created a positive selection score for a pathogen in a target population. In order to assess significance, a resampling procedure was used (Part B in S1 Fig). The first step was to randomly choose a gene in the target population that did not have evidence of interaction with the target pathogen. Step 2 was to filter SNPs in the gene for LD, as described. Each SNP contained a positive selection score that was computed from the target population. The SNP with the median positive selection score was removed and added to set B*, which also started as an empty set (step 3). This step was repeated until the number of SNPs from the randomly chosen gene matched the number of SNPs in the target pathogen or until there were no more SNPs in the randomly chosen gene (step 4a). If the number of SNPs in set B* (referred to as |B*|) did not equal the number of SNPs representing the target pathogen (referred to as |B|), the process was repeated by randomly choosing another gene (step 4b). Once |B*| matched the number of SNPs representing the target pathogen, they were summed to produce a random neutral positive selection score in the target population. This score was compared to the actual score, as they were both a sum of independent SNPs and were of equal size. The main difference was that one set of SNPs was associated with the target pathogen and the other was randomly chosen. Pathogens with fewer than 5 chosen median SNPs were discarded. Twenty-six diseases remained out of the original 151 after applying this filtering procedure.

### Detection of selection in a single population

As specified in step 6 in Part A in S1 Fig, the positive selection score of a pathogen in a population is computed by summing all SNPs in genes whose products interact with the pathogen. Let ***B*** represent this set of SNPs after filtering for LD and let b_i_ represent the i^th^ SNP *B*. The positive selection score for a pathogen in a single population was computed as follows.

For b_i_ a single SNP, let ***iHS(***b_i_***)*** denote the iHS score of that SNP.

For **B** a set of SNPs, let ***iHS(*B*)*** denote the sum of the iHS scores of the SNP in **B**.

$$iHS(B)=\sum_{i}^{\left|B\right|}iHS(b_{i})$$

The value ***iHS(B)*** represents the positive selection score for a pathogen in a population. In order to assess whether ***iHS(B)*** was larger than expected by random chance, we modeled the distribution of the ***iHS*** function when applied to a set of SNPs of the same size as ***B***. We generated 2,000,000 neutral positive selection scores for the target pathogen to provide an expected distribution and compute a p-value for the observed positive selection score using the method described in Part B in S1 Fig. The p-value for ***iHS(B)*** was obtained by computing a positive selection score for 2 million randomly generated “neutral pathogens” (i.e. a pathogen that interacts with human proteins that exhibit randomly assigned selection scores). More explicitly, a “neutral pathogen” refers to a set of randomly chosen human genes that would represent a pathogen failing to exert selective pressure if they were to interact with a pathogen. The number of times that the randomly generated positive selection scores were greater than the observed positive selection score was used to create a p-value for the null hypothesis of no selection.

Let ***I*** represent the indicator function that returns 1 if true and 0 if false. The probability that a randomly generated neutral pathogen X will have a greater cumulative ***iHS*** value is shown below.

B^*^_i_ is the collection of SNPs chosen for the i^th^ “neutral pathogen”

$$P(X>iHS(B))=\frac{1+\sum_{i=1}^{2,000,000}I(iHS({B^{*}}_{i}>iHS(B))}{1+2,000,000}$$

### Detection of selection across multiple populations

A distinct p-value was produced for each pathogen/population pair. We used a Kolmogorov-Smirnov (KS) test on the set of 53 p-values (one for each population) associated with each pathogen to test for deviation from a uniform distribution. We used a one-sided KS test because only pathogens associated with lower p-values across worldwide populations would indicate the presence of positive selection. The expected proportion of false positives for a p-value (q-value), for each pathogen was computed using the Benjamini-Hochberg method [71, 72]. The “effect size” for a pathogen was computed by taking the mean effect size of the pathogen across all populations.

### Analysis of shared selection signatures across populations

We investigated whether the same genes in all worldwide human population were under selection for the infectious diseases studied. There are some differences in SNP coverage across different populations. In addition, the iHS score cannot be computed reliably in SNPs with low allele frequencies. For these reasons, it is not always possible to assign an iHS score to the same SNPs across all populations. As a result, some SNPs in our data set occur in some populations, but are absent in others. When assessing the commonality of human genes undergoing selection across two populations, only human genes covered in our data set for both populations were included. Each gene’s iHS score was defined as the mean iHS score for all SNPs in the gene. We checked for a correlation between positive selection scores of human genes interacting with the target pathogen in the 15 most significant populations as determined by the population’s p-value for selection in human genes interacting with the target pathogen. Kendall’s rank correlation was used to assess whether these two iHS scores were correlated across all shared genes interacting with a pathogen. Correlation describes extent of common genes that underwent selection in different populations.

### Enrichment analysis for HIV GWAS

Enrichment analysis was applied to investigate whether the genes in pathogen-interaction networks and under positive selection were also associated with HIV risk and host control. For this analysis, the maximum absolute iHS value in any population was utilized for each SNP. To test for enrichment, p-value cutoffs were chosen for eight published HIV GWAS datasets [36–43]. Enrichment analyses were based on Fisher’s exact test, and fold enrichment was calculated based on observed vs. expected overlap. The total number of SNPs was based on the overlap between the Illumina HumanHap550 chip (commonly used in HIV GWAS) and the set of independent HIV-interactome SNPs referred to as Set B in Methods above.

## Supporting information

S1 Fig Project pipeline. **A**: All SNPs within genes producing proteins that exclusively interact with the target pathogen are isolated using the combined host-pathogen PPI database. A set of SNPs that are not in LD are chosen to represent the positive selection impact the target pathogen has imposed on a specified population. **B**: A randomization approach produces a null distribution for the iHS impact score generated in the preceding step. **C**: SNPs with in LD are removed when computing each pathogen’s positive selection score in a target population and when producing the randomized (neutral) impact score with respect to a specific pathogen. The SNP with the median iHS is plucked/retained. Removal of the SNP with the median iHS is followed by removal of all SNPs in LD in the surrounding region. This process repeats until all SNPs have either been plucked/retained or removed due to being in LD with a plucked/retained SNP. Many randomized impact score are computed to generate a null distribution for the impact score from step **A**.
(TIF)

S1 Table Infectious diseases exhibiting signs of positive selection in the 53 human genome diversity panel populations were probed for selection in the 3 populations found in the HapMap II data set. *Yersinia pestis*, Zaire Ebola virus, and the measles virus exhibit a p-value < 0.05 in the European derived and East Asian populations, respectively (highlighted in red).
(DOCX)

S2 Table The p-value represents the probability of surpassing the observed mean iHS value under the null hypothesis of neutral selection. The effect size represents the mean |iHS| in all SNPs found within genes that interact with HIV-1.
(DOCX)
