# Supplementary figures and images for: Systematic detection of positive selection in the human-pathogen interactome and lasting effects on infectious disease susceptibility
Source: PLoS One. 2018 May 25;13(5):e0196676. doi: 10.1371/journal.pone.0196676 (PMC5969750; doi:10.1371/journal.pone.0196676)

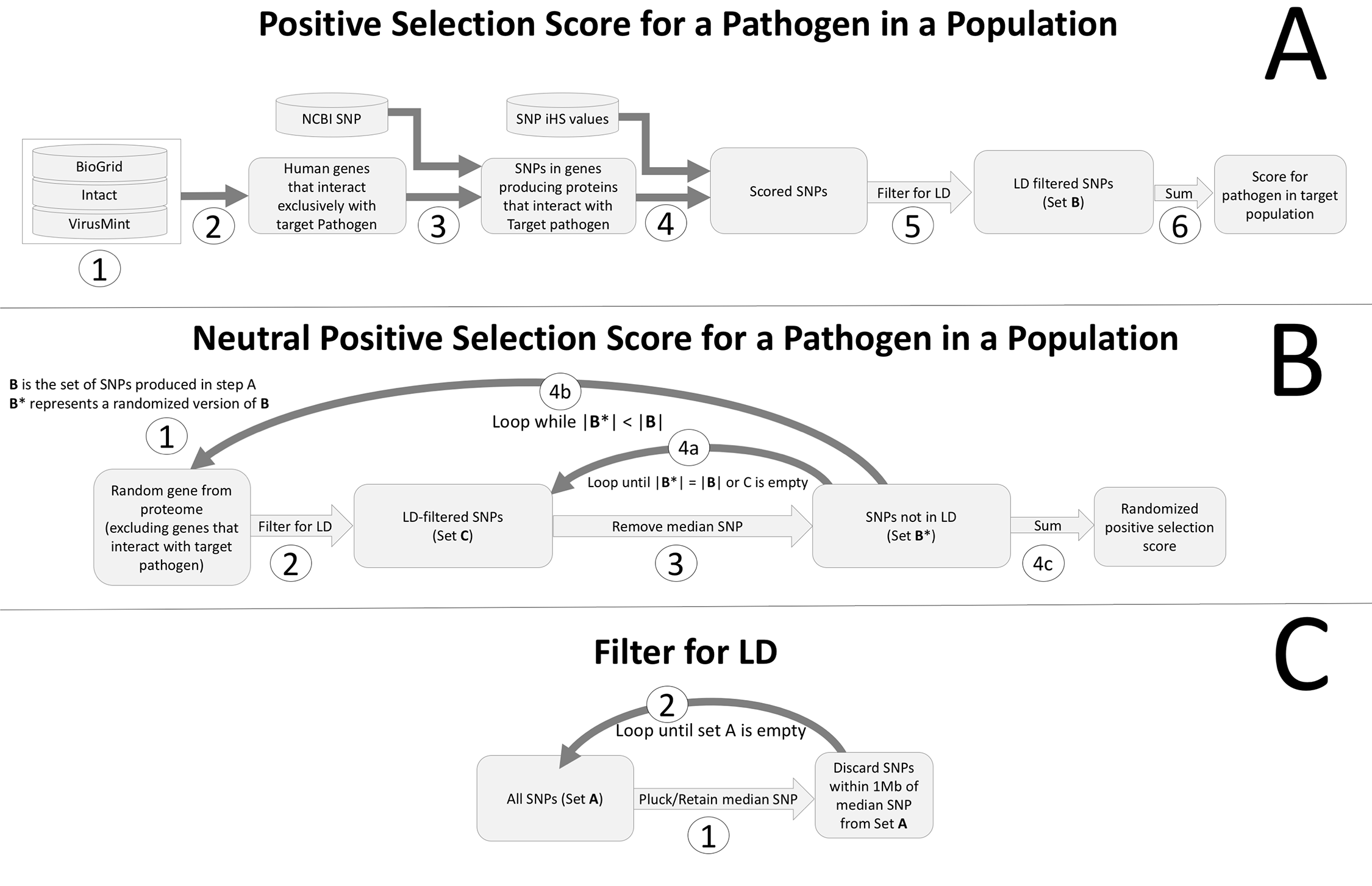

Supplement: S1 Fig — A: All SNPs within genes producing proteins that exclusively interact with the target pathogen are isolated using the combined host-pathogen PPI database. A set of SNPs that are not in LD are chosen to represent the positive selection impact the target pathogen has imposed on a specified population. B: A randomization approach produces a null distribution for the iHS impact score generated in the preceding step. C: SNPs with in LD are removed when computing each pathogen’s positive selection score in a target population and when producing the randomized (neutral) impact score with respect to a specific pathogen. The SNP with the median iHS is plucked/retained. Removal of the SNP with the median iHS is followed by removal of all SNPs in LD in the surrounding region. This process repeats until all SNPs have either been plucked/retained or removed due to being in LD with a plucked/retained SNP. Many randomized impact score are computed to generate a null distribution for the impact score from step A. (TIF) [file pone.0196676.s001.tif]
