# Supplementary material for: Systematic detection of positive selection in the human-pathogen interactome and lasting effects on infectious disease susceptibility
Source: PLoS One. 2018 May 25;13(5):e0196676. doi: 10.1371/journal.pone.0196676 (PMC5969750; doi:10.1371/journal.pone.0196676)
Supplement: S1 Table — Yersinia pestis, Zaire Ebola virus, and the measles virus exhibit a p-value < 0.05 in the European derived and East Asian populations, respectively (highlighted in red). (DOCX) [file pone.0196676.s002.docx]

## S1 Table. Pathogen selection in 3 HapMap populations

| Population | Disease | Tax ID | Effect Size | P-Value | SNPs |
| --- | --- | --- | --- | --- | --- |
| **Europe** | Yersinia pestis | 632 | 0.742 | 0.021 | 491 |
|  | HIV-1 | 11676 | 0.724 | 0.205 | 259 |
|  | Zaire Ebola virus | 186538 | 1.024 | 0.048 | 6 |
|  | Francisella tularensis | 263 | 0.697 | 0.575 | 209 |
|  | Dengue virus | 12637 | 0.878 | 0.078 | 14 |
|  | Human resp. syncytial virus | 11250 | 0.678 | 0.626 | 40 |
|  | Measles virus | 11234 | 0.690 | 0.590 | 72 |
|  | Rubella virus | 11041 | 0.612 | 0.857 | 26 |
|  | Bacillus anthracis | 1392 | 0.740 | 0.0514 | 346 |
|  | Yersinia pestis | 632 | 0.726 | 0.290 | 492 |
|  | HIV-1 | 11676 | 0.689 | 0.870 | 264 |
|  | Zaire Ebola virus | 186538 | 0.894 | 0.148 | 6 |
|  | Francisella tularensis | 263 | 0.718 | 0.482 | 215 |
| **Africa** | Dengue virus | 12637 | 0.770 | 0.296 | 15 |
|  | Human resp. syncytial virus | 11250 | 0.715 | 0.509 | 41 |
|  | Measles virus | 11234 | 0.597 | 0.997 | 73 |
|  | Rubella virus | 11041 | 0.782 | 0.207 | 27 |
|  | Bacillus anthracis | 1392 | 0.698 | 0.816 | 351 |
| **East Asia** | Yersinia pestis | 632 | 0.715 | 0.172 | 490 |
|  | HIV-1 | 11676 | 0.685 | 0.660 | 251 |
|  | Zaire Ebola virus | 186538 | 0.666 | 0.533 | 6 |
|  | Francisella tularensis | 263 | 0.690 | 0.582 | 208 |
|  | Dengue virus | 12637 | 0.742 | 0.330 | 14 |
|  | Human resp. syncytial virus | 11250 | 0.678 | 0.626 | 40 |
|  | Measles virus | 11234 | 0.815 | 0.016 | 72 |
|  | Rubella virus | 11041 | 0.689 | 0.522 | 27 |
|  | Bacillus anthracis | 1392 | 0.683 | 0.718 | 347 |

Infectious diseases exhibiting signs of positive selection in the 53 Human Genome Diversity Panel populations were probed for selection in the 3 populations found in the HapMap II data set. *Yersinia pestis*, Zaire Ebola virus, and the measles virus exhibit a p-value < 0.05 in the European derived and East Asian populations, respectively (highlighted in red).
