# Supplementary material for: Systematic detection of positive selection in the human-pathogen interactome and lasting effects on infectious disease susceptibility
Source: PLoS One. 2018 May 25;13(5):e0196676. doi: 10.1371/journal.pone.0196676 (PMC5969750; doi:10.1371/journal.pone.0196676)
Supplement: S2 Table — The effect size represents the mean |iHS| in all SNPs found within genes that interact with HIV-1. (DOCX) [file pone.0196676.s003.docx]

## S2 Table 2. HIV-1 selection across worldwide populations

| **#** | **Region** | **Population** | **Effect Size** | **P-Value** | **SNPs** |
| --- | --- | --- | --- | --- | --- |
| 1 | Central South Asia | Burusho | 0.775 | 4.24x10^-3^ | 247 |
| 2 | Africa | Mbuti Pygmy | 0.757 | 1.06x10^-2^ | 228 |
| 3 | East Asia | Yi | 0.763 | 1.26x10^-2^ | 234 |
| 4 | East Asia | Mongola | 0.753 | 1.46x10^-2^ | 236 |
| 5 | Central South Asia | Pathan | 0.751 | 2.13x10^-2^ | 245 |
| 6 | East Asia | Yoruba | 0.746 | 3.10x10^-2^ | 243 |
| 7 | East Asia | Xibo | 0.780 | 3.53x10^-2^ | 237 |
| 8 | Africa | Bantu South Africa | 0.740 | 3.62x10^-2^ | 240 |
| 9 | East Asia | Naxi | 0.770 | 3.85x10^-2^ | 233 |
| 10 | America | Maya | 0.746 | 7.90x10^-2^ | 233 |
| 11 | East Asia | Lahu | 0.751 | 9.40x10^-2^ | 235 |
| 12 | Central South Asia | Brahui | 0.733 | 9.66x10^-2^ | 246 |
| 13 | Central South Asia | Balochi | 0.731 | 9.90x10^-2^ | 247 |
| 14 | East Asia | Oroqen | 0.755 | 0.101 | 232 |
| 15 | East Asia | Dai | 0.715 | 0.107 | 237 |
| 16 | America | Surui | 0.776 | 0.108 | 168 |
| 17 | Central South Asia | Hazara | 0.734 | 0.108 | 245 |
| 18 | Europe | Orcadian | 0.731 | 0.134 | 243 |
| 19 | Middle East | Bedouin | 0.725 | 0.161 | 245 |
| 20 | America | Karitiana | 0.741 | 0.176 | 197 |

The p-value represents the probability of surpassing the observed mean iHS value under the null hypothesis of neutral selection. The effect size represents the mean |iHS| in all SNPs found within genes that interact with HIV-1.
